# HIV/AIDS, SARS, and COVID-19: the trajectory of China’s pandemic responses and its changing politics in a contested world

**DOI:** 10.1186/s12992-023-01011-x

**Published:** 2024-01-02

**Authors:** Yanqiu Rachel Zhou

**Affiliations:** Department of Health, Aging & Society, McMaster University, 1280 Main Street West, L8S 4M4 Hamilton, ON Canada

**Keywords:** Pandemic response, China, Global health governance, Politics, Globalization

## Abstract

The COVID-19 pandemic has revealed the contested politics of global health governance, though we still don’t know enough about the dynamics of domestic pandemic responses, or about the relationship between the politics of those responses and the politics of global health governance, both of which have changed significantly in recent decades. Focusing on three cases (HIV/AIDS, SARS, and COVID-19) of cross-border infectious diseases, this article explores the trajectory of China’s pandemic responses in the context of globalization. Attending to changing politics at domestic, international, and global levels, I argue that those responses have been a complex combination of China’s domestic politics (e.g., priorities, institutions, leadership, and timing), its international relations (especially with the US), and its engagements with global health governance. It is concluded that the increasing divergence of pandemic responses in a time of ubiquitous global health crisis demands urgent attention to the connections (including contestations) between domestic pandemic responses and the evolvement of global health governance from a broader perspective that considers changes in geopolitics.

**Supplementary Information:**

The online version contains supplementary material available at 10.1186/s12992-023-01011-x.

## Introduction

On May 5, 2023 the World Health Organization (WHO) Director-General declared that COVID-19 was no longer a Public Health Emergency of International Concern (PHEIC). By that time over 765 million confirmed cases (with over 6.9 million deaths) had been reported globally [1]. They include over 99 million confirmed (with over 0.12 million deaths) in China, and over 103 million (with over 1.1 million deaths) in the United States (US) [2, 3]. The global scale of the pandemic, including the size of the gap between reported cases in the two superpowers, suggests a complex tale of globalization and public health, in which the relationships among the major actors of global health governance – in particular, the US, the WHO, and China – have rapidly evolved against the background of contemporary globalization processes. Although the COVID-19 pandemic has revealed the contested politics of global health governance [4–6], we still don’t know enough about the dynamics of domestic pandemic responses, or about the relationship between the politics of those responses and the politics of global health governance, both of which have changed significantly in recent decades.

Focusing on the trajectory of China’s pandemic responses in the context of globalization, this article explores three cross-border infectious diseases – HIV/AIDS, SARS, and COVID-19 – that constitute important moments in this country’s engagement with global health governance. Offering a review of the related literature, I attend closely to the relationship between the politics of domestic public health responses and the politics of global health governance in a changing world. This article’s inevitably limited scope precludes consideration of the 2009 H1N1 pandemic, which, in any case, was politically less contentious than the other three in view of its timing, sociocultural meanings (e.g., ‘not a Chinese virus’), and milder impacts in China [7, 8].

The first section examines China’s engagement with international collaborations on HIV/AIDS in a post-1989 context in which what it calls ‘stability maintenance’ has become the government’s top priority. Its balancing act between international HIV/AIDS securitization and domestic political stability has also constrained the role of civil society in shaping future public health governance. The second section sheds light on the response of China, an emerging economy, to SARS as an epidemic with potential for global spread and as ‘the first post-Westphalian pathogen’ [9] that constituted an enormous challenge to the traditional state-centric governance framework of Westphalian public health. The key principle of the Westphalian system is sovereignty: that is, each state reigns supreme over its territory and people, even when it comes to international cooperation on infectious diseases [9]. The SARS outbreaks also motivated the Chinese government to introduce an infectious disease *direct* reporting system, and the WHO to revise its International Health Regulations (IHR) in 2005. The third section explores how the COVID-19 response of China, now an economic superpower, has been further complicated by the sharpening geopolitics (i.e., US-China rivalry) at the current phase of globalization. The inflexibility of China’s zero-COVID policy in the face of the rapid mutation of the virus illustrates the limits of its approach that relies on a centralized political system in the context of global health crisis.

Attending to changing politics at domestic, international, and global levels, I argue that the trajectory of China’s pandemic responses has been a complex combination of domestic politics (e.g., priorities, institutions, ideology, leadership, and timing), international relations (especially with the US), and its engagement with global health governance. Although both the HIV/AIDS pandemic (focusing on the 1990s and thereafter) and the SARS outbreaks in 2002–2003 facilitated China’s participation in global health governance, its zero-COVID strategy to the end of 2022 suggests a controversial direction. It is concluded that the increasing divergence of pandemic responses in a time of ubiquitous global health crisis demands urgent attention to the connections (including contestations) between domestic pandemic responses and the evolvement of global health governance from a broader perspective that considers changes in geopolitics.

## HIV/AIDS: international cooperation in the contexts of ‘health security’ and ‘stability maintenance’

China’s first detected HIV case – in an American tourist – was identified in 1985, four years after the WHO set up its office in Beijing. Its first phase (1985–1988) involved imported cases in coastal cities: mostly foreigners and overseas Chinese [10]. In the earlier period of the pandemic, HIV/AIDS in China was largely constructed or demonized as a ‘Western disease’, or a disease resulting from a ‘Western’ lifestyle (e.g., sexual ‘promiscuity) [11]. The comprehensive and integrated national response to HIV/AIDS was not developed until the late 1990s; by then international resources (e.g., funding, ideas for better policy and practice, training, technological assistance, laboratory equipment, and infrastructure construction) had started to pour in [12, 13]. The Joint United Nations Programme on HIV/AIDS (UNAIDS) estimated that China could have 10 million people with HIV or AIDS by 2010 [10, 14]. By that time the country had participated in 267 international cooperation projects on HIV/AIDS, and received over US $526 million from over 40 multilateral organizations (e.g., UN agencies and the World Bank), bilateral governmental organizations (e.g., the UK's Department for International Development, the Swedish International Development Cooperation Agency, and the US’s CDC Global AIDS Program), and international non-governmental organizations (NGOs) or foundations (e.g., the Bill and Melinda Gates Foundation and the Clinton Foundation) [12, 15].

In April 2000 the Clinton administration in the US declared HIV/AIDS a major threat to national security, with the potential to ‘topple foreign governments, touch off ethnic wars and undo decades of work in building free-market democracies abroad’ [16]. This security framing was quickly adopted at the supranational level in July 2000 through United Nations Security Council (UNSC) Resolution 1308, which designated HIV/AIDS as comprising ‘a genuine threat to international peace and security’ [17, 18]. Despite the government’s worry about its negative impact on the country’s flourishing trade and tourist industry, in 2001 China became one of the 189 signatories to the UN General Assembly Declaration of Commitment on HIV/AIDS and started to incorporate its discourses and goals into national policy making [19]. Treating it as a strategic issue for social stability, economic development, national prosperity and security, the government made HIV/AIDS prevention and treatment a priority [19]. In 2004 China’s State Council established the HIV/AIDS Working Committee, with the Vice-Premier and the Minister of Health as directors [20]. From 1982 to 2021 the central government issued 471 policy documents to combat the epidemic and most (413 out of 471) were issued after 1995 [21].

Although the securitization of HIV/AIDS can help mobilize resources and encourage high-level state involvement, making it a national security threat drew attention away from other public health issues, legitimatized security actors’ authority to override the human rights of people living with HIV (PLWH), and compromised the ongoing efforts to destigmatize the disease [22–24]. Offering a means of lifting public health above ‘mere politics’ into the ‘high politics’ – that is, issues of vital importance and survival of the state – of international relations, this approach rendered individual states’ narrow national interest in health security incompatible with the solidarity required to tackle a transborder infectious disease threat [25–29]. Along with the debate about the extent to which various UN agencies have meaningfully integrated this framing ever since, securitization of infectious diseases at the WHO has also encountered contestations between the Global North and the Global South, given their asymmetric interests and capacities for enforcement [17, 22].

Among the international funding agencies that emerged after this securitizing move, the Global Fund to Fight AIDS, Tuberculosis and Malaria (hereinafter the Global Fund) was the largest international health cooperation program at work in China; during its 10-year operation there it was active in more than two-thirds of the counties and districts [30]. As the world’s major multilateral funder in global health and a public-private partnership (PPP) to which the US government is the largest single donor [31], since 2003 it has approved $1.81 billion to support China’s fight against the three diseases, including HIV/AIDS [30]. By the end of 2013, however, the Global Fund had officially closed its portfolio in the country. Difficulties raising funds and growing pressures for emerging economies to shoulder more international responsibilities led the organization to make China, as well as several other G20 countries, ineligible for Phase Two renewal of existing grants [19, 30].

China’s response to this pandemic, as well as to related international collaborations, intertwines with its cautious political, economic, and social transition to and integration with a globalizing world. In 1978 Deng Xiaoping, the chief designer of China’s economic reform after the disastrous Cultural Revolution (1966–1976), announced the Open Door Policy, reopening the country to foreign businesses [32]. Its rapidly expanding economic ties with Western countries were, however, tested by the 1989 Tiananmen Student Movement, and not revived until the early 1990s [33]. ‘Stability maintenance (*weiwen* 维稳)’ – including prevention of anti-regime protests – has become the top priority for the Chinese Communist Party (CCP) regime in a post-1989 context, not to mention the cascading collapse of communist regimes in Eastern Europe and the former Soviet Union [34, 35]. As pointed out by Deng [36] when meeting with President George Bush on February 26, 1989: ‘Without a stable environment, we can accomplish nothing and may even lose what we have gained.’

One of the outcomes of China’s international engagement is the rapid growth of HIV/AIDS-focused grassroots NGOs, which were viewed as the most successful civil society group developed in the country [19]. Noting that many NGOs were not registered with the government, Gåsemyr – based on various published materials – estimated that by the end of 2013 the actual number of organizations involved with HIV/AIDS-related issues was around 550 [37]. In light of the low priority of health policies since the economic reform and the generally restricted engagement of grassroots NGOs in China, the high degree of political recognition of and the proliferation of NGOs, solely observed in the area of HIV/AIDS, are largely attributable to the normative and technical effects of its securitization introduced by the UNSC and supported by the Global Fund [19].

The Chinese government’s strategies on NGOs were, nevertheless, a mixture of control and empowerment to forge a path of non-profit development, in favour of health NGOs that are politically inactive but professionally capable [38]. The government encouraged such NGOs to address the gaps in its own public health provision, which were consequences of its neoliberal reforms (e.g., privatization and a fee-for-service system in health care) [19, 39–41]. In practice, the state has not only relied on health NGOs to provide services for stigmatized groups (e.g., sex workers, gay men, and injection drug users) that present the government with legal or ‘moral’ challenges; it has also deliberately encouraged a climate of competition between NGOs and channelled their activities away from broader political and social objectives [19, 42].

Despite its invaluable contribution to the involvement of civil society and strengthening China’s response to this pandemic, the Global Fund’s sudden departure also had profound impacts on the country’s state-society relations. The subsequent huge funding gaps not only compromised the sustainability of the already fragmented HIV/AIDS-focused NGOs; it also made the government a major source of funding in the field and those government-organized NGOs the favoured ones [19, 30]. It is therefore challenging for NGOs that share interests to form alliances to influence policy making, to call for changes in the current political environment, and to foster stronger partnerships between the government and civil society. In effect, according to Lo [19], international HIV securitization was weakened with the rise of the Chinese government’s commitment on HIV/AIDS interventions.

When it comes to international collaboration on HIV/AIDS, since the 1990s China’s relationships with both the US and multilateral organizations (including the WHO) have nevertheless been largely cooperative. With the support of the Clinton administration, in 2001 China joined the World Trade Organization (WTO), and soon became an emerging economy. At that stage of the international pandemic response China, as a newcomer to globalization, was primarily a recipient of international aid (financial, technological, and ideational). Juggling between HIV/AIDS securitization at an international level and ‘stability maintenance’ at a domestic level also meant lost opportunities for the Chinese government to address the persisting deficiencies in its *broader* public health systems and to explore the role of civil society in making domestic public health governance more transparent, in protecting human rights, and in planning for future emergency preparedness [19].

## SARS (SARS-CoV-1): a ‘people’s war’ against ‘the first post-westphalian pathogen’

During the 2002–2003 SARS (severe acute respiratory syndrome) outbreak, the WHO received reports of 8,098 probable cases (with 774 deaths) from 26 countries, with most cases occurring in China, Hong Kong, Singapore, Taiwan, Viet Nam, Canada, and the US [43, 44]. Calling it the ‘first severe and readily transmissible new disease to emerge in the 21st century’, the WHO also explicitly linked it with globalization, as it ‘showed a clear capacity to spread along the routes of international air travel’ [45]. As was not the case with previous infectious diseases, the *rapid* human-to-human transmission of this airborne disease immediately raised questions about the effectiveness of *state-centric* public health responses to a pandemic that does not recognize borders. Calling SARS ‘the first post-Westphalian pathogen’, Fidler explains that it emerged in a new political and governance context, in which approaches (including conceptions, strategies, and attitudes) to public health had been shifting *away* from Westphalian principles guided by national sovereignty and non-intervention since the 1990s [9, 46]. Although post-Westphalian public health (i.e., globalization of public health) began to emerge before the SARS outbreaks, Fidler saw the latter as the first opportunity to evaluate how the new governance approach that requires cross-border coordination and cooperation would succeed against serious microbial attack on a *global* basis [9].

With China being the first epicentre, the Chinese government’s three-month cover-up came under heavy international criticism, including from the WHO [17]. Despite its moral duty, as a responsible emerging economy, to the world to make it public, China’s initial responses did not, strictly, deviate from the Westphalian principles of the primacy of national territory and sovereignty, because it had neither any international *legal* obligation to report it to any state or international organization, nor any express duty to cooperate with the WHO. In Fidler’s view, China’s initial reaction resulted from its failure to grasp the post-Westphalian context of infectious disease governance, or public health’s ‘new world order’ [9, 46].

That said, China’s political structure is also to blame. Reporting an epidemic in its hierarchical bureaucracy – from local physician/hospital to local CDC, provincial health bureau, the provincial government and, eventually, the Ministry of Health – was not only time-consuming, but also highly risky to the career prospects of those in the system. As a novel or unknown disease back then, as well, SARS – ‘atypical pneumonia’ (*feidian* 非典) at the time – was not listed in the law as an infectious disease that local authorities were legally obliged to report [47]. In this light, the delay or hesitation at each level of the bureaucracy was not a complete surprise but a result of individual and political calculations. When the news finally reached the central government, the timing was problematic, given the impending National People’s Congress (NPC) in March 2003, which represented the highest-level power within the party-state and, hence, political sensitivity in China’s domestic politics. Under international pressure from Western news media, thanks to a whistleblower (Jiang Yanyong, a retired doctor at a military hospital), in April 2003 the Chinese government and, in turn, Chinese mass media finally addressed the issue [47, 48]. The earlier cover-up, of course, turned out to be a serious miscalculation.

It is important to note that SARS emerged about one year after China’s entry into the WTO; at almost the same time the new CCP leader, Hu Jintao, came to office (on November 15, 2002). From the perspective of the new leadership that was attempting to consolidate power, therefore, SARS represented ‘the most severe socio-political crisis for the Chinese leadership since 1989’, and the SARS response directly concerned ‘the health and security of the people, overall state of reform, development and stability, and China’s national interest and international image’ [49]. China’s response to SARS was highly politicized after Hu’s (whose presidential tenure lasted from 2003 to 2013) declaration of a ‘people’s war’ against it on April 14, 2003 [50]. While this war metaphor seemed characteristic of authoritarian China, its connection with the notion of health security has become clear, given its wide use (including in the US and Germany) during the later COVID-19 pandemic [22, 51].

With SARS now a top priority of the government, responses came directly under the leadership of the Vice-premier Wu Yi, who was also made the new health minister; inspection teams were sent by the State Council to the provinces, and slack government officials were penalized [47, 50]. A strategy analogous to that of the ‘patriotic health movement’ of the Mao era was adopted, relying on mass mobilization and faster coordination of resources, communication, and collaboration across sectors (including mass media), places, and hierarchies of governmental systems [50, 52]. The party-state’s tremendous capacity to mobilize political zeal and resources was demonstrated by the spectacle of a lightning-fast construction project in late April 2003: it took only seven days for China to build a 1000-bed *fangcang* (方舱) hospital – or ‘square-cabin’ hospital – for SARS patients in a northern suburb of Beijing, at a cost of 160 million yuan (over US$19 million) [53]. With its ostensible success, China’s approach to the SARS crisis – reflecting the archaic modes of governance characterized by mass mobilization, authoritarian control from the centre, and the uncompromising use of police and, even, military power [54] – *was repeated* during the COVID-19 pandemic nearly 17 years later.

The shorter duration and smaller scope of China’s SARS response meant that many of its aspects, including the states’ long-term fiscal incapacity and the unsustainability of highly restrictive disease control measures in the context of weak social protection, had not yet been put to the test. It was also unclear whether the response that heavily relied on the CCP’s political system was adequate to prevent and contain future infectious disease outbreaks, given the number of problems remaining, such as underfunded public health systems, the absence of genuine civil society engagement, and the backlash of human rights violations in the name of disease control [9, 47, 54]. To overcome its bureaucratic obstacles, in 2004 the government initiated an Internet-based disease reporting system (covering 37 diseases across all areas of the country) that allows local hospitals to *directly* report suspected cases to the China CDC and the Ministry of Health [55, 56]. Nevertheless, the Chinese government faced many other challenges relating to social determinants of health, including the negative effects of economic globalization on hundreds of thousands of urban workers and poor farmers in the contexts of increased competition and low prices, and of neoliberal welfare reforms on accessibility of healthcare systems, especially in rural areas [57, 58].

At the level of global health governance, SARS was often seen as a ‘watershed’ moment, because it revealed the inadequacy of health security as a rhetorical device at the WHO, the conflict between ‘post-Westphalian public health governance’ and national sovereignty, and the need for a globalist approach to cross-border infectious disease threats in the context of globalization [17, 47, 59, 60]. After March 2003 the WHO issued an unprecedented series of global health alerts and emergency travel advisories (e.g., postponing travel to Beijing and Toronto, as SARS affected areas) without the authorization of the concerned countries, which ‘marks an important securitizing move’ at a supranational level [17, 61]. The normative conflict between China and the WHO during the SARS crisis over the precedence of sovereignty or, conversely, global health security also facilitated the WHO’s revision of the IHR in 2005, representing changes in norm and approach to global health cooperation [62, 63]. In this light, SARS has triggered a ‘new way of working’ on infectious diseases, constituting part of ‘a victory for the emerging framework of post-Westphalian public health’ [9]. As a legally binding international agreement the IHR requires states parties to detect, assess, report, and respond to public health events, though in reality most lower-income countries lack that capacity [64]. Moreover, many barriers to global health governance still exist, such as insufficient research and development (R&D) on the coronavirus (SARS-CoV) and inadequate compliance of nation-states with obligations under the IHR ensuing from a lack of awareness, capacity, or motivation [65–68].

It is worth noting that until the Trump administration SARS also facilitated expanding scientific cooperation on disease control between the US and China. These two countries’ collaboration was very extensive, for example, in research and development (R&D) during both the SARS and Ebola outbreaks [69, 70]. Many of the younger, well-educated staff of China’s new CDCs, hired after SARS, as well as the Chinese health experts dispatched to fight Ebola in West Africa in 2014–2015, benefited from being trained by and working with American colleagues [71]. China has also collaborated internationally with global health governance actors (including the US and the WHO) on other infectious diseases, such as H1N1 in 2009 and H7N9 in 2013, though the tension between global health security and state-centric public health responses has remained [72–74].

## COVID-19 (SARS-CoV-2): the zero-COVID policy in the context of US-China rivalry

Many substantial changes have taken place in the landscape of global health governance over the past decade. Since the SARS outbreaks China has embraced multilateralism in health governance and started exerting increasing influence on the WHO [75, 76]. China’s contributions have grown by 52% since 2014 to approximately $86 million in 2019 in part due to its economic development [77]. Since the Trump administration halted its funding to the WHO in early 2020, China has committed $30 million and pledged more [78, 79]. As well, health diplomacy (e.g., its Ebola intervention in West Africa and vaccine diplomacy during the COVID-19 pandemic) has been integral to China’s Belt and Road Initiative (BRI), which was introduced when President Xi Jinping came to power in 2013, three years after it became the world’s second-largest economy [80]. As a China-led global infrastructure development strategy involving more than 150 countries and international organizations, BRI also challenges the US-led global architecture [81, 82]. Seeing a rising China as its strategic competitor and a threat to its global hegemony, the US’s policy on the country has also turned. The growing rivalry is exemplified by developments ranging from the trade war before the COVID-19 pandemic to the narrative battles and vaccine race during it [83–85, 68]. In its ‘Reality check of US allegations against China on COVID-19’, for instance, the Chinese Ministry of Foreign Affairs also pointed out that ‘the US was the first country to pull out personnel from its consulate-general in Wuhan and the first to announce entry restrictions on all Chinese citizens’ [86].

The Chinese government’s report to the WHO on the novel coronavirus (*xinguan* 新冠) was faster than its SARS report. Days after the central government *learned of* the outbreak in Wuhan in late December 2019, the WHO was informed in early January 2020, though the virus’s generic sequence was not released until January 12 [87]. That said, there has been some controversy about whether the timeline of China’s notification to the WHO has met its obligations under Article 6 of the IHR (2005) [88]. While international criticism of the Chinese government’s initial delayed response has concentrated on its suppression of information, the failure of the aforementioned infectious disease direct reporting system – a post-SARS technical innovation to overcome bureaucratic hurdles and achieve faster reporting by the frontline health workers – is also noticeable [89]. In addition to the political calculations of local officials and governments in a top-down performance evaluation system, the barriers to an early warning include a salient lack of other monitoring mechanisms (e.g., organic civil society and free media) in a context of intensified state control during Xi’s era [90–92]. Although Li Wenliang – one of the doctors who posted warnings on social media in late December 2019, and who died of COVID-19 on February 7, 2020 – was later mainstreamed by the central government as a ‘hero’ of the CCP system [86], the continuing reliance on whistleblowers to break bad news also indicates the inadequacy of implementing a self-claimed ‘world-class’ technological infrastructure *alone* to accelerate disease reporting in China’s political environment.

China began taking immediate action after President Xi’s public remarks on January 20, and its zero-COVID (*qingling* 清零) policy lasted from January 2020 to December 2022 [93, 94]. Within three months of the outbreak in Wuhan (the first epicentre) and in nearby infected areas, the virus was quickly managed by implementing such disease control measures as rigorous lockdowns, travel restrictions, mobile hospitals, and quick coordination of national resources (e.g., daily necessities and medical teams) [95, 96]. After this first wave, China’s pandemic response was further articulated as the ‘dynamic zero-COVID’ (*dongtai qingling*动态清零) policy and became routinized country-wide. Unlike the traditional containment and mitigation strategies, as Liu et al. explained in China CDC Weekly, its core is ‘to take effective and comprehensive measures to deal with localized COVID-19 cases precisely, to quickly cut off the transmission chain, and to end the epidemic in a timely manner (to “find one, end one”) [97]’. The elimination strategy involves a combination of nucleic acid testing, data-driven surveillance technology (e.g., for contact tracing), border screening, mandatory quarantine, community screening, and other control measures [96–98]. The high level of integration of digital surveillance and big data in pandemic responses has generated concerns about privacy and the expansion of state power, which is not unique to China [98, 99]. Residential neighbourhoods’ (*shequ* 社区) crucial role in disease control and social governance, and the reliance on them for public health policy enforcement, have also renewed the CCP’s power at a grassroots level, and constrained neighbourhood autonomy in private governance in the long run [100, 101].

Before Shanghai’s lockdown in 2022, the effectiveness of the zero-COVID policy was avidly exploited by the state to bolster the CCP regime’s legitimacy at both domestic and international levels [102–104]. Despite the restrictions imposed on people’s daily lives, the disease control measures were tolerated by most, and even helped to engender public trust in local governments [105]. By March 2021 China had approved five domestic COVID-19 vaccines – including the two that were soon approved by the WHO — for emergency use; all can be kept at normal fridge temperatures, a big advantage over the Western alternatives, especially in lower-income countries with logistical challenges to vaccine storage and transport [106, 107].

While the rest of world was struggling with the multiple waves of the pandemic, China quickly returned to normal economic activities. According to the WTO, for instance, in 2020 China became the largest exporter of COVID-19 critical medical products (with a value of US$ 105 billion) and was the second largest exporter – after the EU – of COVID vaccines, with a cumulative share of over 32 per cent of worldwide exports (totalling over 1.9 billion doses) as of May 31, 2022 [108, 109]. Amid the spread of the Omicron variant, China held the 2022 Winter Olympics in February by using a closed-loop (*bihuan* 闭环) management system, which separated the Chinese public from a parallel world of international participants [110]. In state media, China’s successful containment of COVID-19 was framed as proof of the superiority of its political system (over Western liberal democracies) and of the advantages of authoritarian governance (e.g., centralized decision making, coordinating all national activities, and collectivism) in the context of emergency management [110–112].

After the recurrence of COVID-19 outbreaks in other mega-cities (e.g., Shenzhen, Guangzhou, Beijing, and Xi’an), however, the two-month *city-wide* lockdown of Shanghai (from April 3 to May 31, 2022) further evidenced the double-edged nature of the politics of the zero-COVID policy: when its benefits and costs were impossible to balance, the policy also set traps in which the government risked losing the public’s trust and support [103, 113]. The closure of this global financial hub and China’s wealthiest city, with 25 million residents, drew international attention to the policy’s collateral damages and social costs, such as serious shortages of daily necessities (e.g., food and medicines), inaccessibility of non-COVID healthcare, loss of employment and income, mental health deterioration, infringement on human rights, and a significant drop in the city’s GDP [85, 93, 113]. While Shanghai, as a first-order, province-level, administrative unit, had been known for its own effective and minimally invasive strategy of ‘Precise Prevention and Control (*jingzhun fangkong* 精准防控)’, the implementation of lengthy, stringent, ‘medically unnecessary’ lockdowns begs a bigger question [93]: why did China’s zero-COVID policy persist, despite the virus’s changing nature (i.e., Omicron was highly transmissible, less virulent, and possibly asymptomatic), the end of similar policies by other countries, and the warnings of the International Monetary Foundation (IMF) and the WHO about its sustainability [114–116]?

Directing attention to China’s domestic politics, Keng et al. argue that the ‘Xi factor’ was not solely responsible, and multiple systemic constraints together obstructed the country’s decision-makers in adjusting the zero-COVID policy [93]. In an emergency requiring urgent action, for example, the governments – including local governments – tended to conservatively adopt ‘a proven approach’ to minimize political risks [93, 117]. As well, the nature and political structures of the party-state make it easier than in many countries for policymakers to switch between ‘routine administration’ and ‘crisis management’ without incurring strong social resistance. Moreover, the COVID policy represents a critical timing of the political contestation before the 20th National Congress of the CCP, which was postponed from March to October [93]. At this congress the president succeeded in extending his tenure to an unprecedented third term, and advocated a ‘Chinese way of modernization’ (*zhongguoshi xiandaihua* 中国式现代化) as an alternative to Westernization; and Li Qiang, a Xi loyalist who oversaw the stringent COVID controls in Shanghai, was named to the new Politburo Standing Committee, the core circle of power in the ruling CCP [118–120]. In the context of the China-US rivalry, furthermore, China’s pandemic responses became a site of ‘ideological contestations’ and of demonstration that ‘China was the solution [to] and not the cause of the pandemic’ [112, 121]. Here also is a partial explanation of the ill-prepared, abrupt, end of the zero-COVID policy in December 2022, when scattered anti-lockdown protests were transforming into a national and transnational ‘White Paper movement’, and thus posed a serious threat to the legitimacy of both the pandemic policy and the regime [122, 123].

A direct consequence of the U-turn in China’s pandemic response was widespread infection. According to the estimate of Wu Zunyou of China’s CDC in January 2023, since the reopening up to 80 per cent of the Chinese population could have become infected [124, 125]. The finale of China’s pandemic response suggests an impasse for ‘parallel worlds’: that is, in a highly integrated and interconnected world China cannot remain immune to global contagion through an unsustainable elimination strategy. Confronting a rapidly evolving, multi-waved, multi-sited, and lengthy pandemic, China’s top-down approach, which relied heavily on its highly centralized decision-making power and mass mobilization, exposed its significant limitations, such as institutional rigidity, marginalization of the health-scientific community, personnel fatigue, fiscal unsustainability, urban-rural inequalities in accessing health care, and lack of transparency [93, 96, 126]. On an international level China’s lengthy zero-COVID policy also risks perpetuating the ‘new Cold War’ framing that portrays China as a ‘black box’ [127].

## Conclusion

Together these three cases help to sketch *the trajectory* of China’s responses to cross-border infectious diseases, which intersected with domestic politics (e.g., priorities, institutional systems, leadership, and timing), geopolitics (especially US-China relations), and the politics of global health governance in the context of *ongoing* globalization processes. Over the course of that trajectory, China’s relationships with the US, the WHO, and the world have substantially changed. As *a poor, socialist country* new to global society, China’s international engagement with HIV/AIDS after the 1990s was both exploratory and selective, cautiously balanced between opening its economy to the world and maintaining its own sovereign autonomy and socio-political stability in a post-1989 context. The securitization of HIV/AIDS has, on one hand, elevated China’s political responses to the disease and its capacity for international cooperation; on the other, its prioritization of stability maintenance also means a highly mediated role for civil society and international NGOs in its public health governance.

During the SARS crisis, as *an emerging economy* China demonstrated its capacity to quickly contain a novel infectious disease and its willingness to cooperate with the US and the WHO as a leader of global health governance, while struggling with many challenges (e.g., information transparency and equitable access to healthcare resources) resulting from its authoritarian political systems and neoliberal healthcare systems. The successful ‘people’s war’ against SARS in China also helped legitimatize the public health approach that relies on the centralized CCP political system. The successful handling of SARS outbreaks by the WHO’s global campaign was viewed as a victorious start for a post-Westphalian or global health governance, which was seriously tested 15 years later when a truly global-scale pandemic *simultaneousl*y hit both higher- and lower-income countries.

By the time the COVID-19 pandemic occurred, China had become *an economic superpower* and a major player – not just one of the actors – in global health governance, competing with the US to assert its own influence on the world, as well as with the WHO [128]. While China’s domestic politics, as well as its insistence on state sovereignty, has always been integral to its responses to cross-border infectious diseases, its intersection with geopolitics has become sharper and more entrenched during this pandemic against the background of US-China rivalry [129]. While China’s lasting zero-COVID strategy can be understood as a result of its path dependency of policy response, it also suggests the diminishing influence of international actors (including the WHO), as well as of civil society, on the country’s pandemic response.

When it comes to global health governance, what distinguishes China’s COVID-19 response from those it had to HIV/AIDS and SARS is the changing US-China political dynamics [71]. Although the adverse impacts of superpower calculations on international health cooperation were nothing new, unprecedentedly this pandemic itself has become the battleground for these two countries’ competition for power and influence [26]. The intensified geopolitical rivalry has also exacerbated the challenges faced by the WHO as a supranational body with a mandate to direct and coordinate emergency responses and contributed to its marginalization at the early stage of the COVID-19 pandemic [130]. As ‘the most important global health crisis since the 1918 influenza pandemic’ [131], the scope, speed, length, and different waves of the COVID-19 pandemic have revealed both the practical irrelevance of its IHR as a *depoliticized* legal framework for global health cooperation and the constraints of individual states’ – including both China’s and the US’s – healthcare systems [4, 132]. It also reveals that the implementation (including its form and extent) of global health governance ultimately depends on action by nation-states and is thus inevitably conditioned by domestic politics [4]. Meanwhile, the floundering nationalist pandemic responses (e.g., vaccine nationalism) across political regimes have revealed the disconnect between existing global institutions and the dire need for collective action, as well as the complex role of geopolitics – that is, ‘how the distribution of power among states in the international system, including changes in the balance of power, affects state behavior’ [26] – in pursuing global solidarity in an interdependent, yet divided, world [68, 133, 134].

Looking ahead, the dynamics of globalization, as well as of global health governance, will become even more complicated, because the contestations between China and the US are likely to continue, because of, for example, their ‘misplaced ideological hostility’ and the fragility of global supply chains, at the heart of the latter of which is China [135, 136]. In this light, China’s trajectory of pandemic responses in the contexts of globalization and changing geopolitics are also a cautionary tale about why we must attend to the gaps between global health governance agendas and domestic political processes, and find ways to navigate the contestations of national interests on public health issues between states (especially powerful states) in order to foster a collective capacity across differences (e.g., based on economy, political system, and ideology). After all, uncontrollable risks, not limited to pandemics, and potential catastrophes neither respect state boundaries nor are clearly tied to one actor or source in this interconnected ‘world risk society’ [137].

### Electronic supplementary material

Below is the link to the electronic supplementary material.

Supplementary Material 1

## Data Availability

Not applicable.

## Abbreviations

BRI: Belt and Road Initiative
CDC: Center for Disease Control and Prevention
CCP: Chinese Communist Party
Global Fund: Global Fund to Fight AIDS, Tuberculosis and Malaria
IHR: International Health Regulations
IMF: International Monetary Foundation
NGOs: Non-governmental organizations
NPC: National People’s Congress
PHEIC: Public Health Emergency of International Concern
PLWH: People living with HIV (PLWH)
PPP: Public-private partnership
R&D: Research and development
SARS: Severe acute respiratory syndrome
UNAIDS: The Joint United Nations Programme on HIV/AIDS
UN: United Nations
UNSC: United Nations Security Council
US: United States
WHO: World Health Organization
WTO: World Trade Organization
