# Supplementary material for: HIV/AIDS, SARS, and COVID-19: the trajectory of China’s pandemic responses and its changing politics in a contested world
Source: Global Health. 2024 Jan 2;20:1. doi: 10.1186/s12992-023-01011-x (PMC10759387; doi:10.1186/s12992-023-01011-x)
Supplement: Supplementary file 1 — Supplementary Material 1 [file 12992_2023_1011_MOESM1_ESM.docx]

**Acknowledgements**

The author wishes to express her appreciation to Dr. Michael A. Stoto and anonymous reviewers of this journal for their insightful comments and to Kristy Yiu for her research assistance. This work was supported by an internal grant from McMaster University, Canada. This paper was presented at the Bayreuth-Kulmbach Workshop on Global Health Politics, organized by Professor Tim Dorlach, at the University of Bayreuth, Germany, August 30-Sept 1, 2023.
